# A Review of Flood Loss Models as Basis for Harmonization and Benchmarking

**DOI:** 10.1371/journal.pone.0159791

**Published:** 2016-07-25

**Authors:** Tina Gerl, Heidi Kreibich, Guillermo Franco, David Marechal, Kai Schröter

**Affiliations:** 1 Helmholtz Centre Potsdam GFZ German Research Centre for Geosciences, Section Hydrology, Potsdam, Germany; 2 Guy Carpenter & Company Ltd., London, United Kingdom; University California Los Angeles, UNITED STATES

## Abstract

Risk-based approaches have been increasingly accepted and operationalized in flood risk management during recent decades. For instance, commercial flood risk models are used by the insurance industry to assess potential losses, establish the pricing of policies and determine reinsurance needs. Despite considerable progress in the development of loss estimation tools since the 1980s, loss estimates still reflect high uncertainties and disparities that often lead to questioning their quality. This requires an assessment of the validity and robustness of loss models as it affects prioritization and investment decision in flood risk management as well as regulatory requirements and business decisions in the insurance industry. Hence, more effort is needed to quantify uncertainties and undertake validations. Due to a lack of detailed and reliable flood loss data, first order validations are difficult to accomplish, so that model comparisons in terms of benchmarking are essential. It is checked if the models are informed by existing data and knowledge and if the assumptions made in the models are aligned with the existing knowledge. When this alignment is confirmed through validation or benchmarking exercises, the user gains confidence in the models. Before these benchmarking exercises are feasible, however, a cohesive survey of existing knowledge needs to be undertaken. With that aim, this work presents a review of flood loss–or flood vulnerability–relationships collected from the public domain and some professional sources. Our survey analyses 61 sources consisting of publications or software packages, of which 47 are reviewed in detail. This exercise results in probably the most complete review of flood loss models to date containing nearly a thousand vulnerability functions. These functions are highly heterogeneous and only about half of the loss models are found to be accompanied by explicit validation at the time of their proposal. This paper exemplarily presents an approach for a quantitative comparison of disparate models via the reduction to the joint input variables of all models. Harmonization of models for benchmarking and comparison requires profound insight into the model structures, mechanisms and underlying assumptions. Possibilities and challenges are discussed that exist in model harmonization and the application of the inventory in a benchmarking framework.

## Introduction

The global increase of flood damage observed during recent decades [1, 2] is a prime mover to improve our understanding of flood impacts and consequences, for developing reliable loss models and efficiently reducing flood risk. Flood loss models–or flood vulnerability models–describe the relationship between hazard intensity metrics such as flood depth, velocity, etc. and a damage ratio that can be translated into a monetary quantity. These relationships constitute a critical component of flood risk analyses and consequently play an important role in the implementation of risk-oriented management approaches as described by legal frameworks such as the EU-flood risk management directive [3]. Flood loss estimation is also important for insurance and reinsurance companies to design insurance products and set appropriate premiums [4, 5], as well as to estimate probable maximum losses to their portfolios, which in turn helps companies and regulators enforce the industry’s solvency requirements.

Using depth-damage curves for the estimation of flood loss dates back to the 1960s [6, 7] and has been progressively accepted internationally as the standard approach for urban flood loss assessment [8, 9]. By now, a large variety of loss models have appeared differing in purpose, structure, and regional focus. Loss modelling in some instances is performed separately per sector, say residential, commercial, industrial, agricultural, etc. and on different spatial scales, where the units of analysis vary from individual elements at risk to aggregated land use units [10]. Furthermore, loss models differ in their damage metric, i.e. the model outcome may be the estimated absolute loss in monetary terms or it may be the relative loss, expressed as a fraction of the total value of the element at risk [11].

Besides the uni-variable depth damage curves, multi-variable loss functions have been developed that consider more variables in addition to water depth for loss estimation, for instance building type, or precautionary measures or impacts due to contamination [12, 13, 14]. The extension of loss models into the probabilistic domain has introduced the possibility to provide quantitative information about model uncertainty [15, 16, 17, 18]. This considerable progress [10, 19] has resulted in a highly heterogeneous landscape of functional forms of increasing complexity to describe flood damage. In the face of considerable uncertainty associated with loss estimates [4, 16, 20, 21] probabilistic models show promise to increase the reliability of loss estimates impacting flood risk assessments [15].

This growth in flood loss assessment methodologies has not always been accompanied by a sound and explicit model validation process that would document and demonstrate how well models perform the kind of task for which they were intended [22]. This lack of rigorous delineation of when and where a certain model should be applied might result in the adoption of models to geographical regions and flood events that differ from the settings for which they were originally designed. Since variations in local characteristics and systems such as the implementation of precautionary measures have a strong impact on outcomes, models often require regional adjustments [15, 23].

Due to the uncertainties arising from these complexities, the reliability and robustness of flood loss models are often hard to assess. Frequent dispersion in the results leads to question their adequacy and validity in their application. Flood loss model benchmarking and validation are therefore becoming increasingly relevant for instance to public authorities in charge of flood risk assessment and management as well as to the insurance industry since regulatory standards in Europe and elsewhere expect companies to “own” their view of risk, i.e. to understand their risk assessment tools and to adopt a critical view to underpin their decisions on capital requirements, reinsurance, and enterprise risk management [24, 25].

The robustness and validity of the flood loss models used in the risk assessment tools by the insurance companies should thus be tested by 1- comparing them with the models described in the scientific literature and 2- identifying whether such alignment is appropriate for the application in mind. As a consequence, the first step towards the benchmarking and validation of flood loss models should be a compilation of all available references from the scientific literature. Similar initiatives were recently made for other perils such as wind and earthquake [26, 27]. While this survey of scientific literature and the classification of flood loss models are instigated by the particular needs of the insurance industry, the authors believe that this taxonomic exercise is a useful contribution to the flood risk community in general.

This work thus presents an inventory of flood loss models, compiled from a review of scientific papers and research reports. The flood loss models are queried and catalogued according to various dimensions including model specification, geographical characteristics, sectors addressed, input variables used, completeness of model validation, transferability and mathematical formulations. The rationale of the review is to provide a basis for the development of model harmonization approaches and finally for model benchmarking. To build this catalogue of vulnerability functions we extract from the studies these functional relationships and analyze the accompanying descriptions regarding their usage, limitations, and scope. From this inventory we commence a discussion on the harmonization of models. Although this step is beyond the scope of this work, a benchmarking framework will eventually require that all or most models can be compared–at least approximately–within a common referential space.

## Methods Used to Build the Inventory

The compilation of the flood loss model inventory is carried out by collecting references that include original work on the development of loss models within a literature review. We focus on fluvial floods, while offering examples of other flood types such as coastal or lake floods. Only models for direct tangible flood losses are considered. Indirect tangible and all intangible damage are excluded. Direct losses occur as a result of the direct physical impact of a flood event while indirect losses occur outside the hazard area in temporal or spatial terms. Intangible losses refer to damage to people, objects and services that are not easily measurable in monetary terms because they are not directly traded in a market [5, 10, 28].

The meta-data compiled in the inventory presented in this paper (see S1 Table) provides details about the general model philosophy in terms of underlying assumptions, regional embedding as well as units of analysis, flood type, input variables required and other model characteristics.

The functional forms themselves have not been included in the paper or in the supplemental material. However, all necessary references are given to lead the reader to the specific formulations, if that were of interest. In most cases, these references are publically available and can be easily retrieved from the literature. In some limited instances, however, some models or model components might be subjected to intellectual property restrictions that may require the reader to ask for explicit permission from the relevant parties in order to access the information.

### Literature review strategy

Our literature research uses the following mixed strategies: browsing, footnote chasing and consultation [29].

Five experts with 60 collective years of experience in the field of flood loss modelling and with an approximate collective track record of 70 publications in the same field representing varied perspectives from the industry and academia, gave rise to the core recommendations and search strategy. Based on the recommendations of these experts, recent, comprehensive review papers and project reports were searched for flood loss model descriptions. This selection included five review papers [9, 10, 22, 28, 30], six scientific papers [13, 14, 20, 23, 31, 32] and five project reports [1, 33, 34, 35, 36]. Flood loss models were extracted from these documents and from their associated references, for which we searched in bibliographic databases using web-based research platforms.

As these papers mostly covered European studies, partly because other regions adopted standard models like in North America, additional references were searched in order to complement and update the inventory of flood loss models. Within this search, terms in English language were used since this is the international language of science. However, non-English references entered the inventory via cross-references in papers and reports of the core recommendations.

The search engines used were the web applications of Science, Sciencedirect, Scopus, and Google or Google Scholar. The searches were carried out in the period from October 2014 to December 2014. The following keywords were searched in the different web search engines using the option ‘search in all fields’ without imposing any date or language restrictions: “flood catastrophe risk model”, “flood damage function”, flood damage curve”, flood damage model”, “flood vulnerability function”, flood vulnerability curve”, flood vulnerability model”, “flood susceptibility”, “flood damage assessment” and “cost of floods”, “losses of floods”, “cost of hazards”, “losses of hazards”. In this process the significance of the publication titles of the first 200 hits for each keyword search sorted by relevance were checked. Relevance of search results was determined by the search engine according to the relative frequency that the search terms used appear in each publication. Next, publication titles were perused and potentially suitable publications were identified for subsequent eligibility assessment. The criterion for eligibility of a publication was if information about a flood loss model function is provided or not. The resulting inventory is the most complete public survey to date.

### Review limitations

The inventory does not represent an exhaustive compilation of all flood loss models, which exist worldwide. The recommendations of experienced experts may have biased the selection as a result of the experts’ own backgrounds and experience. The additional search via web based engines may have introduced an bias towards scientific sources. The flood loss models included in the inventory mostly originate from publications in English although some were extracted from documents in Afrikaans, Japanese, German, Dutch or French which the authors translated with external support. Due to this language bias other existing references e.g. from South- and Central America, China and other countries have not been found. Other loss models, e.g. from commercial tools may not have been accessible.

### Structure of the inventory

The structure of the flood loss model inventory is created on the basis of previous review papers [9, 10, 22, 28, 30]. It contains the following categories: (1) model specification, (2) geographical characteristics, (3) sectors addressed, (4) input variables used, (5) model validation, (6) transferability and (7) model functions. In addition, each category consists of several attributes which are described in Table 1. Category (1) gives general information about the model and its basic concept and application. Furthermore, the database used for model development is identified. Category (2) includes information on spatial scale and extent of the flood loss model, i.e. for which region/catchment the model was built. This category also contains information about the models’ unit of analyses and land use classes as well as the flood type. Category (3) states the sector for which the model was developed. Category (4) specifies the input variables for the flood loss model, concerning flood impact, building characteristics, socio-economic factors and precautionary measures. Category (5) describes if and how the model results are validated. Category (6) describes the feasibility to transfer the model to different regions. Category (7) contains the actual model, i.e. the formula or matrix of flood loss separated for the various considered sectors.

**Table 1:** Structure of the flood loss model inventory.

Category	Attributes	Definition
Model specification	Model	refers to the model *name* and its *abbreviation*
	Reference	*author* name, *year* and publication *title*
	Domain (development background)	information about the publication type, e.g. *scientific paper*, *proceedings*, *thesis*, *report*, or *software manual*
	Approach	model approach type classified according to *empirical (*uses loss data collected after flood events), *engineering/synthetic* (uses loss data collected via what-if-questions), or a *combination* of both types
	Database	*method of data acquisition* for model development, e.g. interview data, building inspections, etc.; including number of cases
	Model type	distinguishes the model according to the number of damage-influencing-factors considered–possible types are *univariate* (assumption that flood damage is influenced only by one factor, mostly the inundation depth) and *multivariate* (flood damage is influenced by multiple factors)
	Model concept	differentiates *deterministic* (no stochastic elements are involved, so the input and output relation of the model is conclusively determined) and *probabilistic* models (multiple results with varying degree of uncertainty are possible due to the implementation of stochastic elements)
	Purpose of model	type of modelled damages, either *insured damages* or *total economic loss*
	Cost base	*replacement costs* (amount it would cost to replace an asset) or *depreciated/repair costs* (cost for the restoration of damaged property)
	Damage metric	model outcomes are *relative* (% of total value) or *absolute* (currency/unit, e.g. €/m²) damage
(2) Geographical characteristics	Geographical scope	regional context of the flood damage model, considering the categories *continent* (e.g. Europe), *country* (e.g. Great Britain), *region/catchment* (e.g. River Thames), *city* (e.g. London)
	Spatial resolution	*global* (i.e. worldwide), *national* (i.e. countrywide), *regional* (part of a country, e.g. county, river catchment), *local* (a a particular place, e.g. city), and *object-based* (e.g. residential house) are the categories of spatial resolution of the model
	Unit of analysis	major entity that is being analyzed, e.g. *individual objects* (single houses) or *aggregated land use classes* (combination of related objects)
	Land use classes	*name of land use data* set and *data source* (e.g. CORINE Land Cover provided by European Environment Agency) as well as *land use classes* (e.g. residential, industrial)
	Spatial category	*rural* (territory outside of a city, i.e. countryside) or *urban* (high-built up areas with high population density, i.e. a city)
	Flood type	flood source considered: *fluvial flood* (water overflows the river banks when surface water runoff exceeds the capacity of channels to accommodate the flow), *flash flood* (flood peak appears within a few hours originating from torrential rainfall), *pluvial flood* (caused by rainfall or snowmelt), *groundwater rise* (water table level rises to the surface level), *coastal flood* (arise from incursion by the ocean), or *dam break* (failing of dikes causes devastating floods)
(3) Sector	Sector	states the sector for which a flood damage function is available, e.g.*residential* (area in which housing is the predominant use, including building and its content, e.g. single-family house), *commercial* (buildings and its content that refer to the exchange of goods and services for money, e.g. bank, retail trade), *industrial* (includes buildings and its content that belong to processing of raw materials and manufacture of goods, e.g. heavy industry), *public/municipal* (institutional buildings used and run by the community, e.g. school, hospital, theatre), *agriculture* (refers to cultivated land for crop production and raising livestock, e.g. vegetables, stables), *infrastructure* (the installation of public transportation system, e.g. roads, railway), *vehicles* (includes cars and other transportation vehicles of private and business customers), *mixed use* (merged class of above mentioned sectors, e.g. industrial/commercial buildings), *others* (e.g. energy & water supply, forest)
(4) Input variables	flood impact	description of the flood event, considering parameters like *water depth*, *inundation duration*, *flood velocity*, *contamination* (e.g. oil), *return period*, *time of flooding*, *recurrence interval*, *distance between object and water front*, *meteorological data* and *intercept*
	Building characteristics	*building type* (e.g. single-family house, high-rise building, factory), *number of floors*, *number of flats*, *floor space*, *construction material* (e.g. masonry, concrete), *age of building*, *heating system* (e.g. district heating, oil), *building quality*, *building value*, *building fragility*, *building content/inventory*
	Socio-economic factors	*household size* (number of persons living in one house), *ownership* (e.g. rental, private), *monthly net income*, *residing period*, *size of company* (nr. employees), *sector of company*, *equipment*, *goods/products/stock*, *crop type*, *gross value*
	Precaution	*precautionary measures* (e.g. flood adapted building structure, mobile flood water barriers), *flood experience*, *early warning*
	Other	*cost of replacement feed/additional costs incurred or saved*, *damage to building* (no building damage or damage)
(5) Validation	Validation	type of model validation, either *qualitative* (cannot be measured with a numerical result) or *quantitative* (numeric estimation of uncertainty)
	Reference	*author* name, *year* and publication *title* should be given
(6) Transferability	Transferability	information if the transferability of the model is *tested*
	Reference	*author* name, *year* and publication *title* describing the transferability of the model
(7) Function	Type of function/matrix	*univariate*, *multivariate*
	Sector	see description of “sectors” in “geographical characteristics”
	Specific unit of analyses	*specific object* (e.g. single-family house) or *land-use class* (e.g. manufacturing)
	Damage function	damage function *formula* and *legend*
	Damage matrix	is expressed in *water depth* and *damage value*. The *legend* describes the units of input variables
	Damage matrix (for agriculture)	consists of *time of flood event*, *damage value* and *flood duration*

## Observations on the Flood Loss Model Inventory

Within the literature review we identified 66 publications from the search in web search engines. Another sixteen records were identified from the consultation of experts. Footnote chasing of the recommended review and original research papers as well as project reports contributed a number of nine additional project reports. After removal of duplicate publications, 89 records were retained for further analyses. All 89 records were perused in order to check for eligibility to be included in the flood loss model inventory. Only those publications were retained which provide information about the flood loss model function. This step lead to the exclusion of 28 records (see S1 Fig, S2 Table and S1 Text for a list of excluded references), so that 61 publications were included in the qualitative synthesis. Since, fourteen publications provided redundant information about flood loss models functions finally 47 references are included in the flood loss model inventory.

In this section, we summarize our main observations with regards to the main traits used in the flood loss model taxonomy organized accordingly.

### Model specification (category 1)

**Fig 1:**
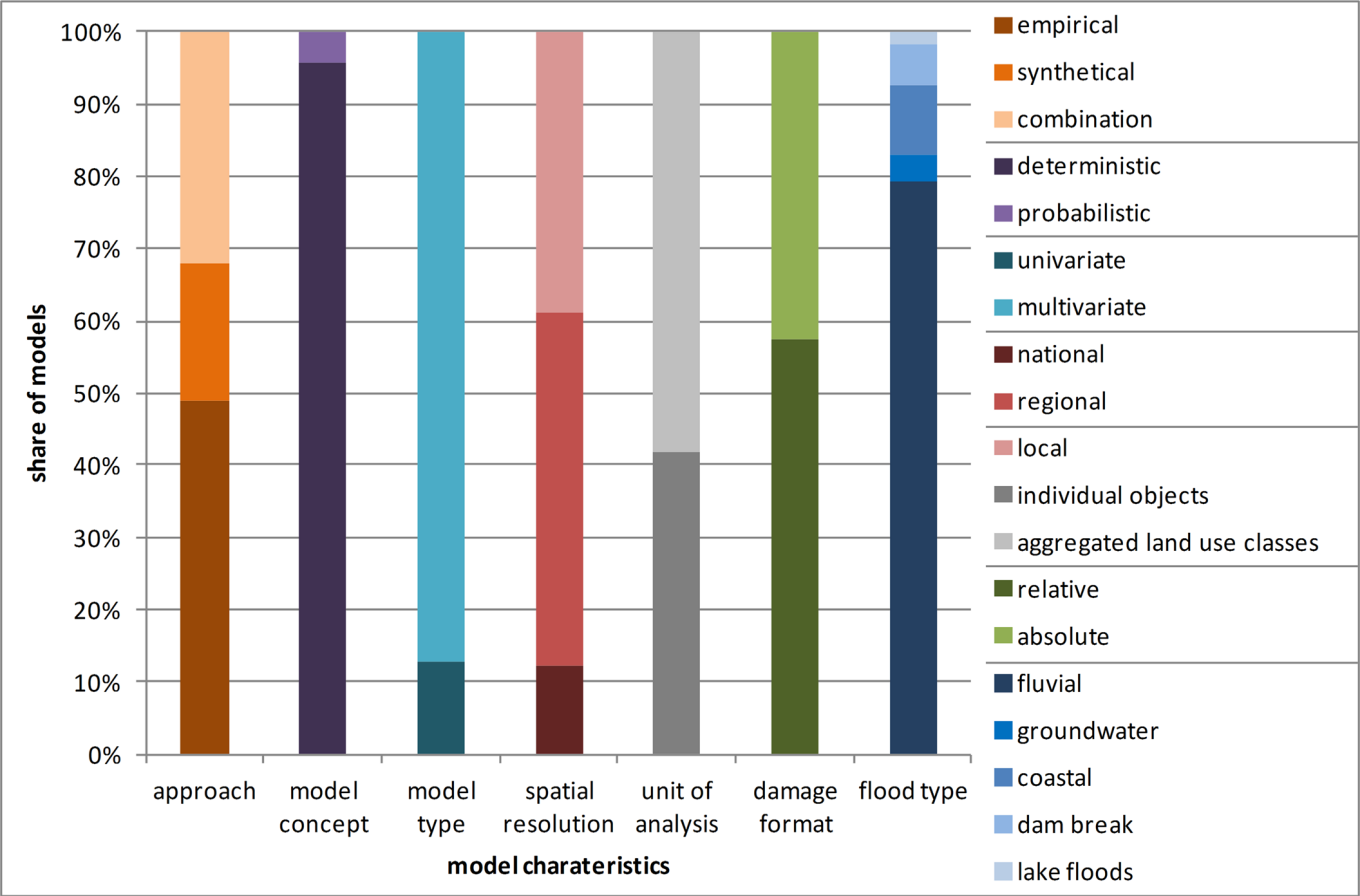
Characteristics of flood loss models contained in the inventory.

An important distinguishing feature for the specification of flood loss models is the model approach or philosophy. There are two approaches that are typically used in developing flood loss functions [37], empirical or engineering/synthetic, as specified in Table 1. A model may also be derived from a combination of both approaches. While the empirical approach uses observed flood damage data collected after flood events, the generation of synthetic damage models is based on hypothetical damage estimates by experts through what-if-analysis (i.e. what is the potential loss if a specific building type is flooded with an inundation depth of 1 meter?). This second approach is often used when detailed empirical damage data are not available or if they are of dubious quality. 49% of the catalogued flood loss models are empirical models while synthetic models account for about 19%. 32% result from a combination of these two approaches (Fig 1).

Model concept, i.e. whether a model is deterministic or probabilistic, is a key attribute. The first type is frequently used to describe the damage processes in terms of a functional relation between flood loss and the variables involved [38]. Models in this category provide point estimates of flood loss. In contrast, probabilistic models provide a distribution of flood loss estimates due to the inclusion of stochastic elements such as the probability of a building being affected by a flood event [12] or the random behavior of certain model parameters or variables [12, 15]. By far the largest share of 96% corresponds to deterministic models versus only 4% of probabilistic approaches. The scarcity of probabilistic models in the literature betrays the rampant epistemic uncertainty and is a strong argument to investigate these models in greater depth in order to leverage their potential to increase the reliability of loss estimates.

We distinguish models depending on whether they use one or many parameters to estimate damages. Uni-variable models are based on the assumption that the response variable, flood loss, is influenced by only one factor, usually the inundation depth, in contrast to multi-variable models which use more than one predictor. Although it is internationally accepted that flood damage is mainly influenced by the inundation depth, this parameter cannot fully explain the damage data variance [10, 39]. Commonly one distinguishes between impact parameters, reflecting specific characteristics of a flood event (e.g. inundation depth, flow velocity, contamination), and resistance parameters that describe the capability of a flood prone object to resist the flood impact, e.g. building type or construction material. The analysis of the flood loss model inventory shows that the most frequently used impact parameters are water depth, inundation duration and flow velocity. Furthermore, some models use contamination, return period and time of flood event as factors influencing damage [15, 40, 41]. In the agricultural sector, the inundation duration and timing of the flood event are particularly important for flood loss assessment [30]. Building characteristics like building type, number of floors, floor space, construction material, building value and building content/inventory are important resistance parameters in flood damage assessment for urban areas [42, 43, 44]. Additionally, the implementation of precautionary measures in order to reduce flood losses is an important factor [45, 46]. Several models consist of a set of uni-variable loss functions which differentiate for example for building or crop type and other impacts of resistance characteristics. Hence, these models eventually do consider multiple variables to estimate flood loss.

Another criterion for distinguishing flood loss models is the damage metric used. Absolute damage functions estimate the loss in monetary units, while relative damage functions express the expected loss as a proportion of the total asset value of an element at risk. 57% of the flood loss models are relative and 43% are absolute models. The advantage of relative loss models is the better transferability in space and time due to the independence from the local economic setting. On the other hand, information on object assets is required for the estimation of monetary damage. While this is not necessary for absolute damage functions, these models require a regular recalibration. Furthermore, since absolute loss models are developed for a particular study area, it is difficult to transfer them to other regions [10, 47].

### Geographical characteristics (category 2)

**Fig 2:**
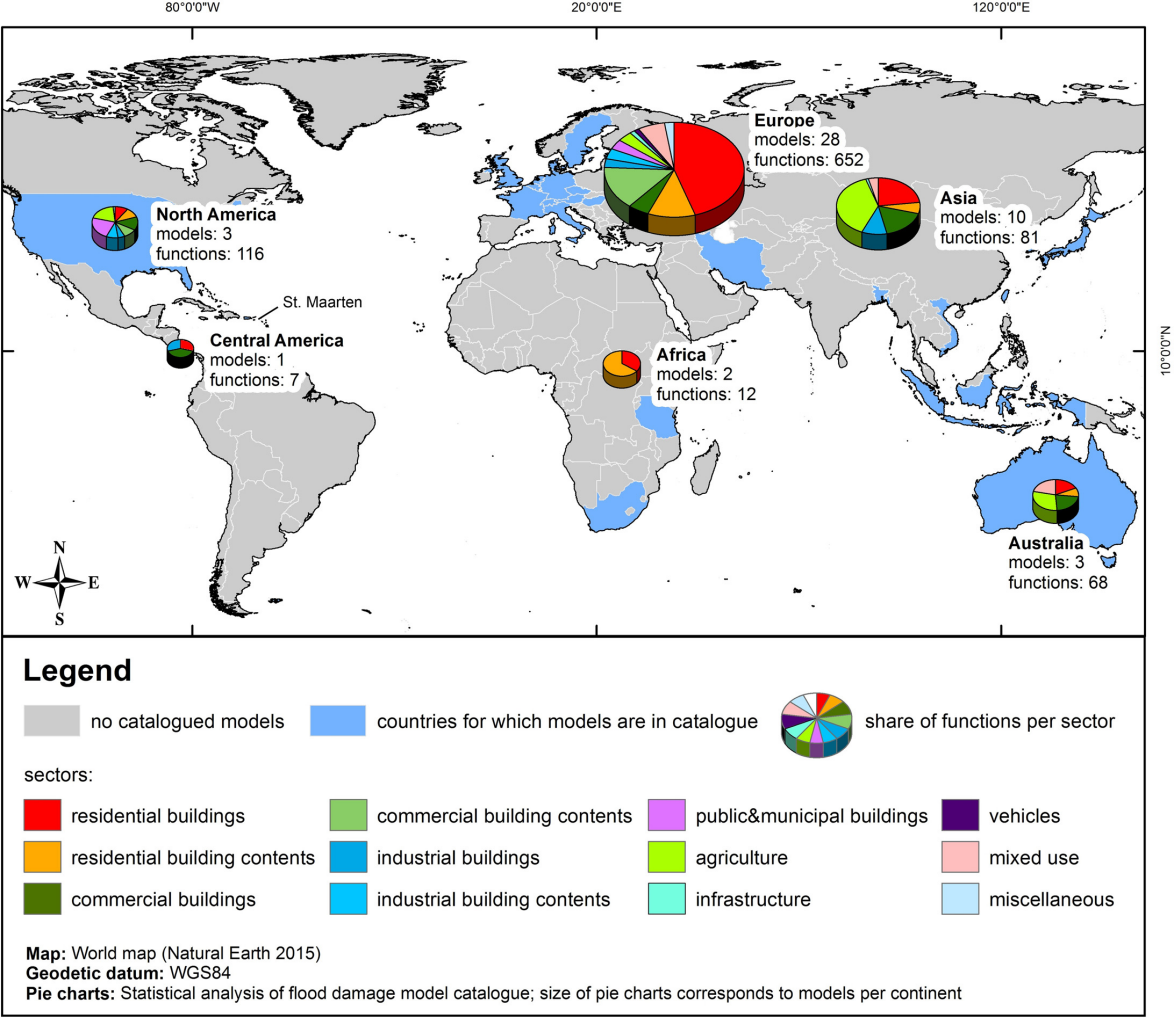
Global distribution of flood loss models and functions for different sectors contained in the inventory.

The inventory contains 47 flood loss models that are distributed across 23 countries (Fig 2). Most of them arise from Europe (60%), followed by Asia (21%), North America (6%), Australia (6%), Africa (4%), and Central and South America (2%). 40 models or about 85% were published as scientific papers and reports. The remaining seven models originate from proceedings, theses, and software manuals.

The large variety of flood loss models around the world can be explained mainly by variations in the objective of country or regional studies as well as national or regional differences in data availability and level of data precision (spatial and temporal resolution), which may depend on budget or time restrictions [18]. The amount of loss models developed in a country is most likely also influenced by the availability of a standard method like the HIS-SSM model in the Netherlands [48, 49] or the HAZUS model in the US [43, 50].

Flood loss estimation is performed on different scales (spatial resolution/unit of analysis), which is mainly determined by data availability. For national or regional studies aggregated land use classes (e.g. residential buildings, commerce) are the norm. In smaller investigation areas (local or object-based scale) the integration of spatial high-resolution land use data with information about individual buildings is more common [51]. On this scale building types are often differentiated by building age, construction material or floor space, for instance. Often separate damage functions are available regarding building structure and building content. The majority of flood loss models are generated to work on regional (49%) and local (39%) scales, i.e. flood damage assessment is performed for entire catchments or urban sprawls. About 12% of the loss models are applicable on a national scale. All in all, 58% consider aggregated land use classes and 42% use individual objects as the unit of analysis.

Due to our search focus on riverine floods, most of the flood loss models contained in the inventory refer to fluvial floods with low flow velocities (79%). Some specialised models focus on estimating losses that occur through dam breaks (6%), groundwater rising (4%) or coastal floods (9%). One model in the inventory tackles lake-flood induced losses [52]. Most studies focus on urban areas, because the largest damages are expected in cites. In contrast, flood losses in agricultural regions are usually much lower [30].

### Sector (category 3)

The flood loss model inventory contains 936 flood loss functions although not all loss functions that belong to a model are included in the inventory. For example, the Multi-Coloured Manual (MCM) [53] and HAZUS-MH [43] contain several hundred functions due to the numerous sub-categories of individual sectors. For these models the average loss functions for individual sectors were selected. Dominant land use categories in the inventory are residential buildings (36%) and their contents (13%), commercial/service sector (8%) and accompanying equipment (10%), public and municipal buildings (7%), and industrial buildings (4%) and their contents (3%). The share of damage functions for the agricultural sector is almost 10%. Infrastructure (1%) and vehicles (<1%) have a significantly lower proportion. About 6% belong to mixed and other uses, see Fig 2.

### Input variables (category 4)

The inventory contains 47 loss models covering different sectors in which different damaging processes are important. Accordingly, the loss models include differing types and number of input variables. 94% of the models use, among others, water depth as an explanatory variable for flood loss. The remaining models (6%) utilize inundation duration and time of flood event as impact parameters [40, 54, 55]. The second most utilized input variable is the floor space of a building (51%) followed by number of flats and socio-economic factors (23%). Flow velocity, goods, products, stock, vehicles, crop type and total area of crop cultivation and contamination are used by 17% of the models. Other input variables used by at least 10% of the models are: construction material, building fragility, flood experience, recurrence interval and age of building, Variables describing information about return period, time of flood event, building value, heating system, flood warning etc. currently seem to play a minor role in loss modeling even though their usefulness to explain flood loss has been demonstrated in various studies. For instance flood warning, and the quality of external response in a flood situation has been shown to have a strong impact on loss (e.g. [9, 56, 57, 58, 59]) and return period has been shown to play a role as well [14].

### Validation and transferability (categories 5 and 6)

An important prerequisite for flood loss assessment is the quality and extent of loss model validation. For 45% of the models, estimated flood losses were validated by comparison with observed loss data, often including an analysis of the relevant factors that affect loss. For the rest of the models the evaluation status is rather unknown and the validation process is not explicitly described in the paper or report that contains the model development description. However, validation of these models may have been undertaken and described in other posterior follow-on literature not included in our review. This holds also true for transferability of the models to other regions. While one cannot bluntly assume that validation and transferability assumptions do not exist for roughly half of the loss models found in our review, it is worrisome that this information is not put forward at the time of the development of the model. One cannot help but wonder whether such validation and transferability exercises were tackled at all when the loss model was proposed. This is not a contributor to increasing our confidence in the models that we use for risk assessments.

### Functional form (category 7)

The functional form of the flood loss models varies considerably depending on the concept, the approach, the sector, and the input variables used. The functions extracted from the literature range from constant loss values for different land uses or sectors, respectively, to complex analytical functions and conditional probability functions. The spectrum of analytical functions covers linear, exponential, logarithmic, polynomial, and square root functions as well as non-linear regression equations to describe the relation between the input variables and flood loss. These analytical functions are usually used within deterministic empirical loss models, where the functions are selected and parameters are derived by curve fitting to observations. In contrast, synthetic models are usually provided as value pair matrices (e.g. in MCM [53] and HAZUS-MH [43]).

## An Illustration of Model Diversity

As stated above, the heterogeneity found in existing flood loss models is daunting. To illustrate the challenges that arise when one aims to compare these loss models with one another or with other models external to the inventory, we choose five arbitrary models and depict their characteristics in this section. These models represent different damage metrics, types of function and model concepts (Table 2). The analysis focuses on two particular sectors only, residential and agricultural.

**Table 2:** Characteristics of the selected example models.

model characteristic		example models (Reference)				
		HAZUS-MH for residential buildings [48]	Zhai et al. [12]	BN-FLEMOps [15]	Yazdi & Neyshabouri [60]	Hess & Morris [61]
Sector	Residential	X	x	X		
	Agricultural				x	x
Damage metric	Absolute		x			x
	Relative	x		x	x	
Type of function	Uni-variable	x			x	
	Multi-variable		x	x		x
Model concept	Deterministic	x	x		x	x
	Probabilistic			x		

**Fig 3:**
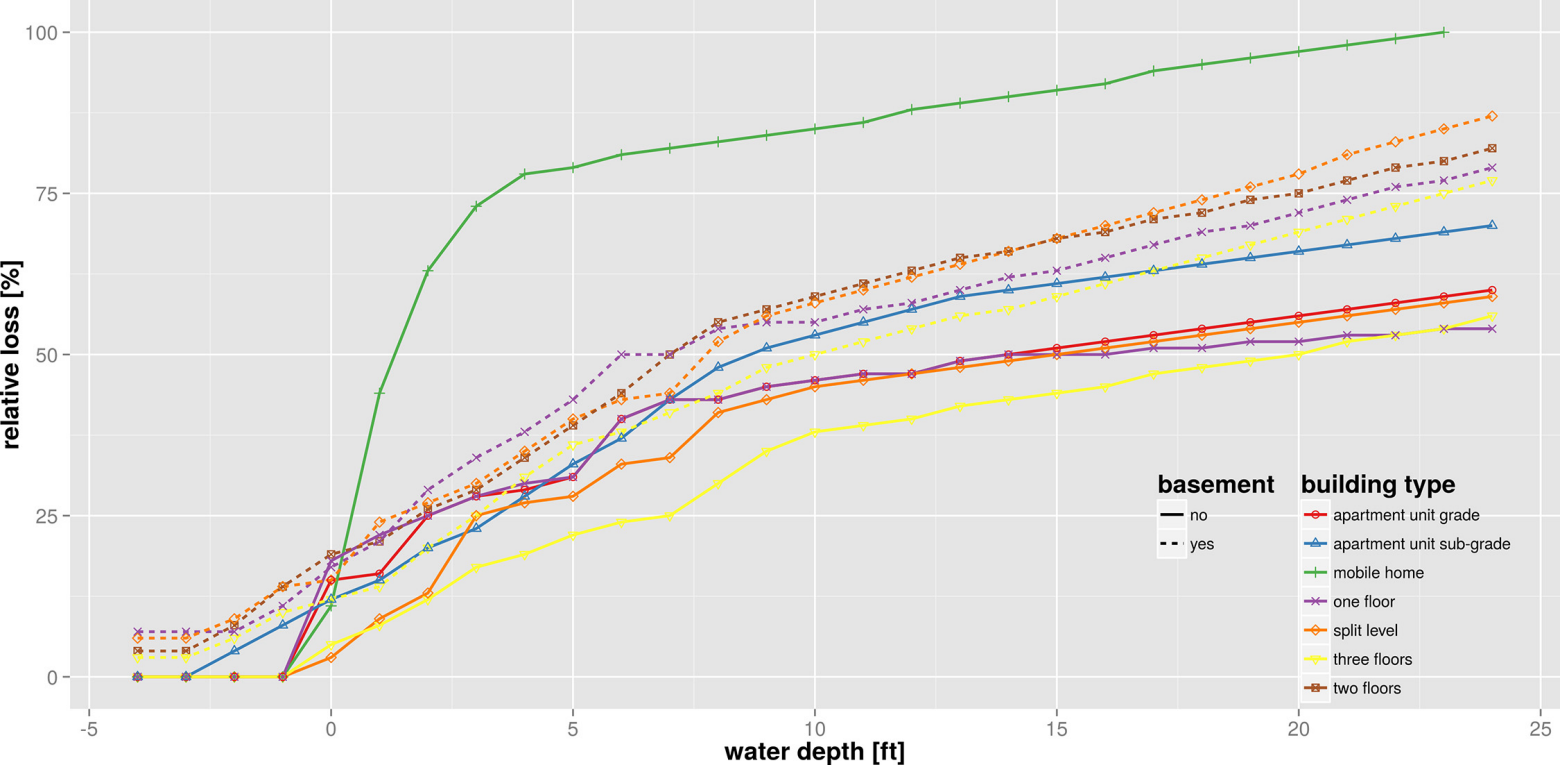
Loss functions of residential buildings in HAZUS-MH [43]; example of a relative, deterministic model using uni-variable loss functions (negative inundation depth refers to inundation in the basement of a building).

The North American deterministic model HAZUS-MH [43] is selected as an example for the residential sector. It contains numerous uni-variable functions that calculate relative flood losses dependent on the inundations depth. The functions are derived based on a combination of empirical and synthetic databases. Individual loss functions are available for residential buildings according to building type, number of floors, and building contents (Fig 3). Loss estimates can be obtained for individual objects; however these values represent average values for a group of similar buildings and hence are usually aggregated for census blocks.

**Fig 4:**
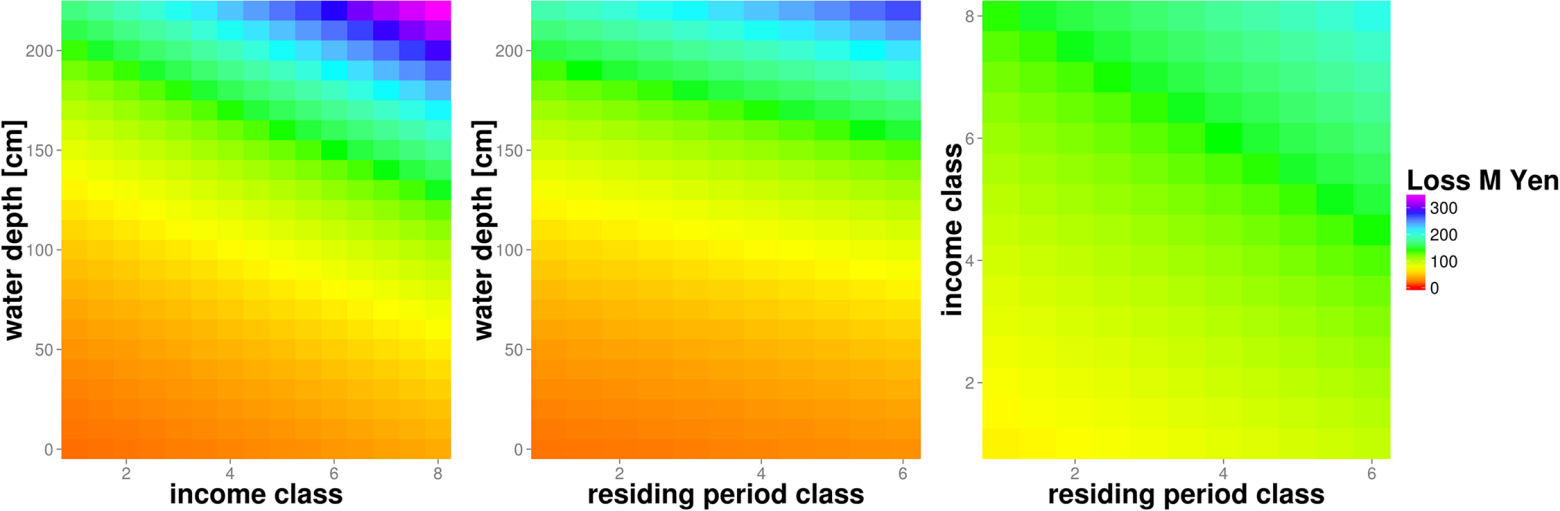
Damage model of Zhai et al. [12] with the damage-influencing factors residing period, income and inundation depth; example for an absolute, deterministic model using multi-variable loss functions.

Zhai et al. [12] derived an empirical deterministic loss function for residential buildings of the megacity of Nagoya in Japan. Considering the loss influencing factors of house ownership, residing period, income and inundation depth this multi-variable model calculates absolute flood losses based on individual buildings. Income is classified into (1) less than ¥3M, (2) ¥3-4M, (3) ¥4-5M, (4) ¥5-6M, (5) ¥6-7M, (6) ¥7-8M, (7) ¥8-10M and (8) >¥10M. Residing period is classified as (1) 1–10 years, (2) 10–20 years, (3) 20–30 years, (4) 30–40 years, (5) 40–50 years, or (6) >50 years. Fig 4 shows three two-dimensional views on the variation of flood loss in ¥M within this multi-variable model space. These sections map water depth and income class, water depth and residing period as well as income and residing period class. For each section the remaining variable is set to its mean value. Loss increases with inundation depth, residing period class and income class. The steepest gradient is observed for increasing water depth in combination with increasing income class and to a lesser extent with increasing residing period class.

**Fig 5:**
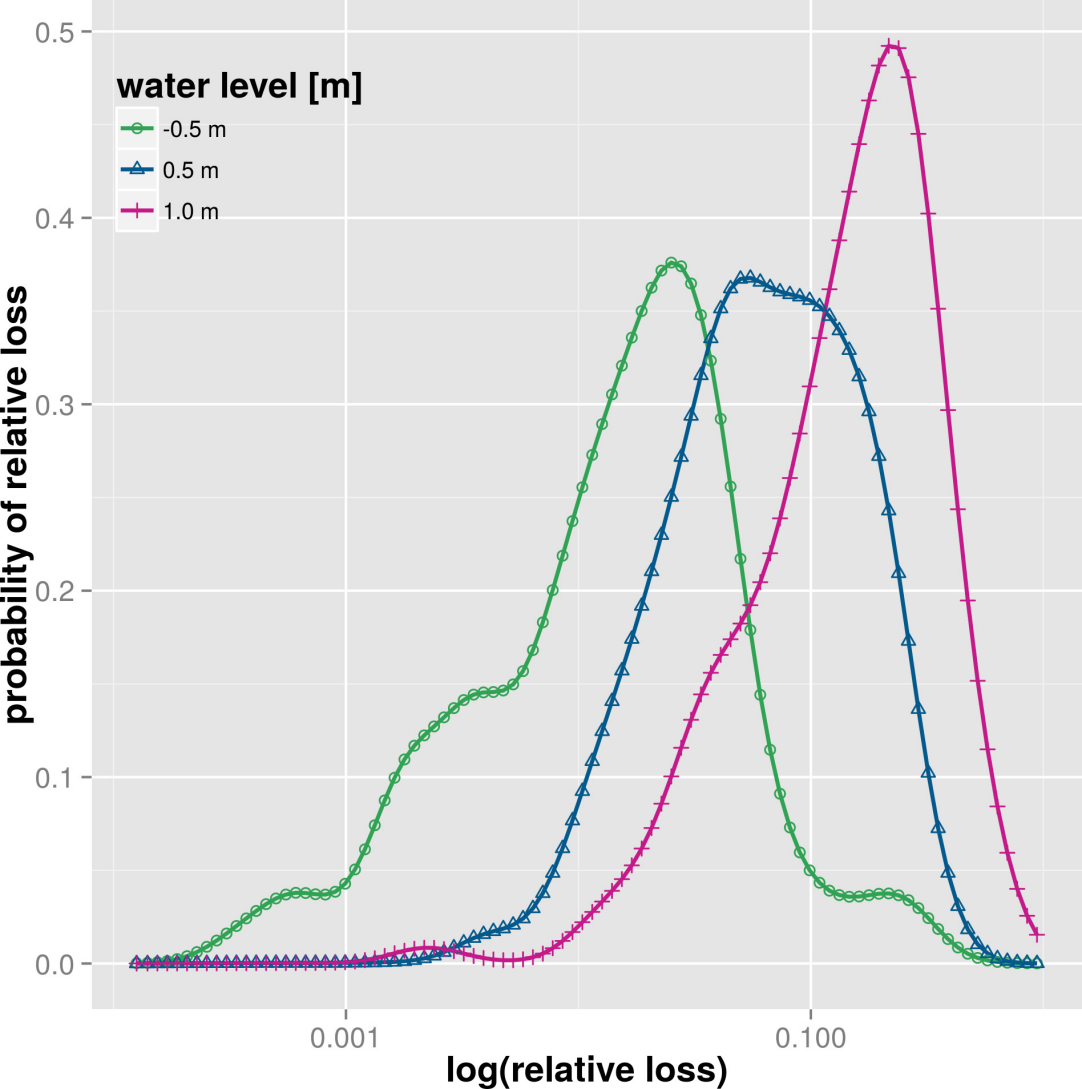
Structure and example distributions of loss estimates for selected water levels of BN-FLEMOps; example for a multi-variable, relative, probabilistic model.

Fig 5 presents the structure of the multi-variable probabilistic model BN-FLEMOps [15] which uses Bayesian Networks to describe the joint probability distribution of the explanatory variables involved. The model was derived from empirical flood damage data for the Elbe and Danube catchments in Germany and can be used to estimate relative loss to residential buildings on the object level. The model considers ten explanatory variables including water depth, contamination, inundation duration, flow velocity, return period, building quality, building value, building type, emergency measures and precaution. On the left in Fig 5 the directed acyclic graph (DAG) of the model is shown which describes the probabilistic independencies of the variables. The DAG, discretization and conditional probability distributions were learned from observed data [17]. The marginal probability distributions of relative loss for varying water levels (-0.5m, 0.5m and 1.0m) but constant observations for precaution and contamination are plotted on the right hand-side of Fig 5. This plot illustrates the shift in probability of relative loss towards higher relative damage with increasing water levels. In contrast to deterministic models the probability distribution of relative loss provides a quantitative estimate of the uncertainty associated with the predicted relative damage.

**Fig 6:**
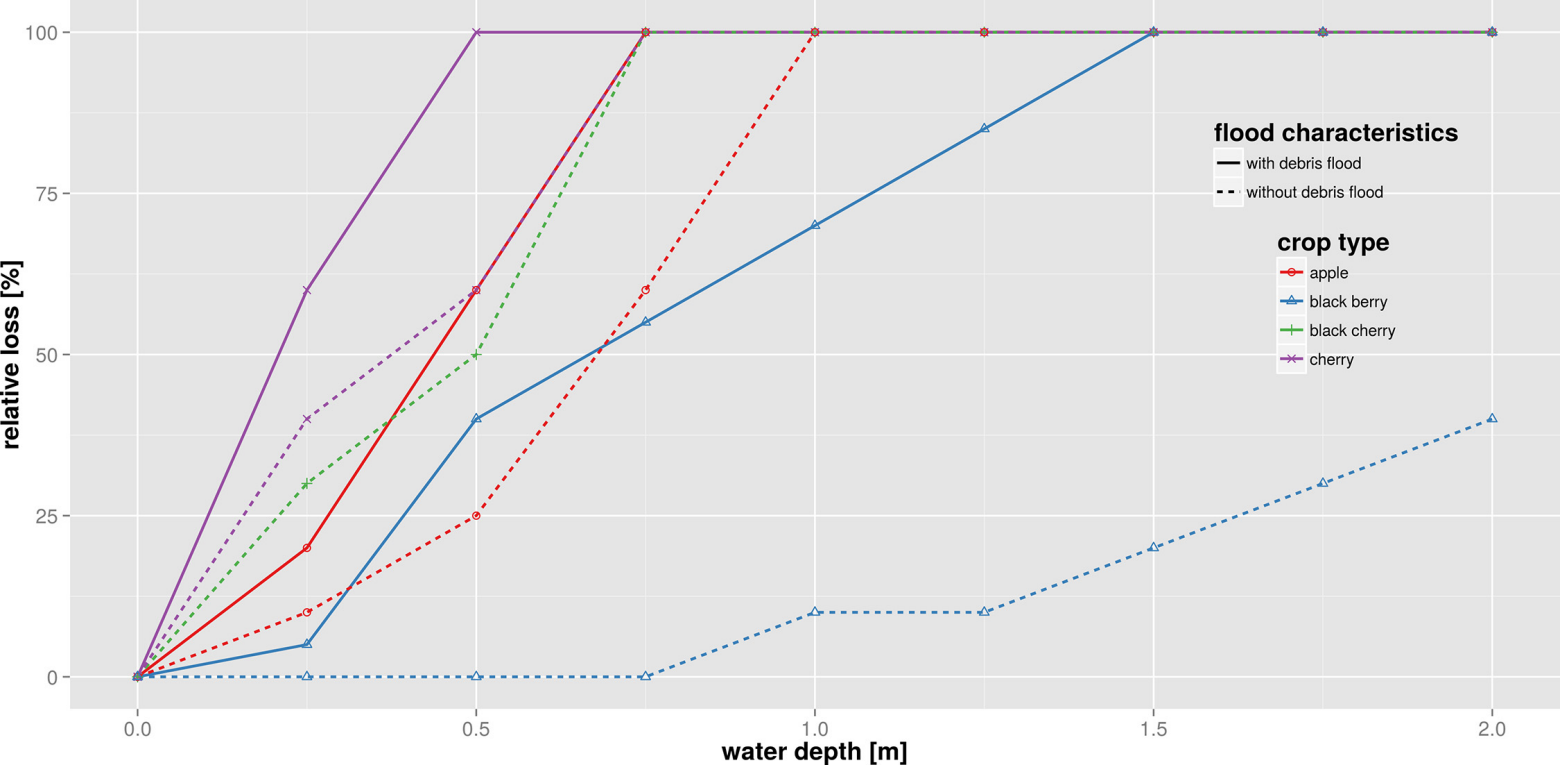
Loss functions of crop types [60]; example for a relative, deterministic model using uni-variable loss functions.

**Fig 7:**
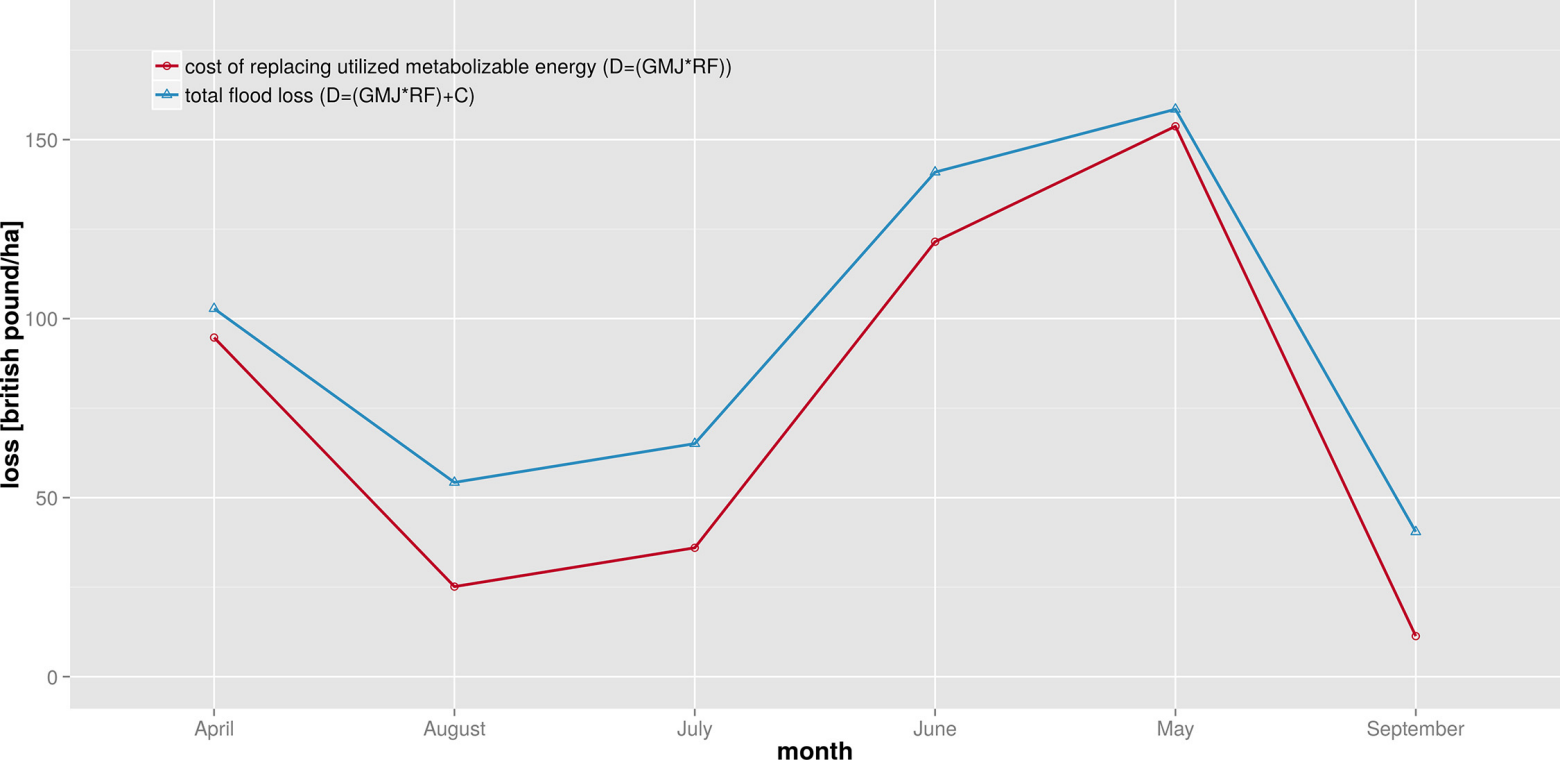
Loss functions for one-cut silage [61]; example for an absolute, deterministic model using multi-variable loss functions. D = total damage [£/ha], GMJ = energy from grass lost due to flooding [MJ/ha], RF = cost of replacement feed [£/ha], C = additional costs incurred (+) or saved (-) [£/ha].

For the agricultural sector the deterministic models of Yazdi & Neyshabouri [60] and Hess & Morris [61] are selected. The first one is developed based on empirical data from the Kan basin in Iran and contains several uni-variable functions for calculating relative flood losses to different crop types (Fig 6). The second model includes a multi-variable loss function for grassland that is based on an empirical-synthetic approach (Fig 7). This function uses information about the energy from grass lost due to flooding (GMJ), cost of replacement feed (RF) and additional costs (C) incurred or saved in order to estimate absolute flood losses in England based on aggregated land use classes.

These examples illustrate typical differences between loss models contained in the inventory which, for the purpose of comparison and benchmarking, need to be harmonized somehow. For instance, models may require different input information in terms of number and type of variables to estimate flood loss. Further, the model outcome might be either in relative or absolute format and might represent loss to varying spatial entities e.g. individual objects or aggregated land use classes.

A strategy to project heterogeneous models onto a common reference space needs to be devised. Otherwise, these models exist in isolation and their performance cannot be easily evaluated against each other’s.

## Challenges in Model Harmonization

Recall that the ultimate aim that instigated this research is to use the inventory of flood loss models as a broad compendium of reference information against which to judge whether commercial flood risk models are reasonably aligned in their assumptions with the existing corpus of science and knowledge. Therefore, the mechanics required to establish comparisons across models are of critical importance.

It is evident, however, in light of the existing heterogeneity of models and approaches that using a common reference frame to carry out sensible comparisons across models is extremely difficult. Devising a strategy that would make this possible in general is well beyond the scope of this paper. Nevertheless, in order to illustrate approaches to tackle this challenge that seem reasonable to the authors at this stage, an exercise is presented in this section to attempt the harmonization of three of the models contained in the inventory.

In this pursuit we ask ourselves whether there exists guidance in the literature as to how to maneuver through this challenge but despite the relevance of this problem, there is no generic procedure available. Therefore, we consider two starting strategies. First, we consider the possibility that all the flood loss models that we wish to compare are transported to a common set of reference variables. In practice this means that for example inundation duration as a flood-impact factor would have to be translated to inundation depth–the most commonly used flood impact variable–in order to compare two models across this dimension. This transformation would ideally provide a set of unique functions using the same common variables. However, due to the heterogeneous nature of the input variables and their ambiguous interrelations and conversion uncertainties, this approach seems tortuous.

As a second option, one could restrict the comparison across flood loss functions to their common intrinsic variables, i.e. the models are driven using only the input variables which are used by all the other models as well. The non-common variables must be set to assume reasonable estimates or a distribution of likely values. In this approach the differences in terms of the number of input variables are maintained and may lead to the comparison of single uni-variable loss functions across models, for instance, differentiating flood loss functions by building types, building material or topographical circumstances. If the number of common variables is relatively low, the dispersion in loss estimates can be expected to be large.

Both approaches aim at harmonizing the dimensionality of uni-variable and multi-variable models and to reconcile differences between the models as regards the model concept (deterministic, probabilistic), their damage format (relative, absolute) and the unit of analysis (individual objects, aggregated land use classes) which may require additional considerations.

**Fig 8:**
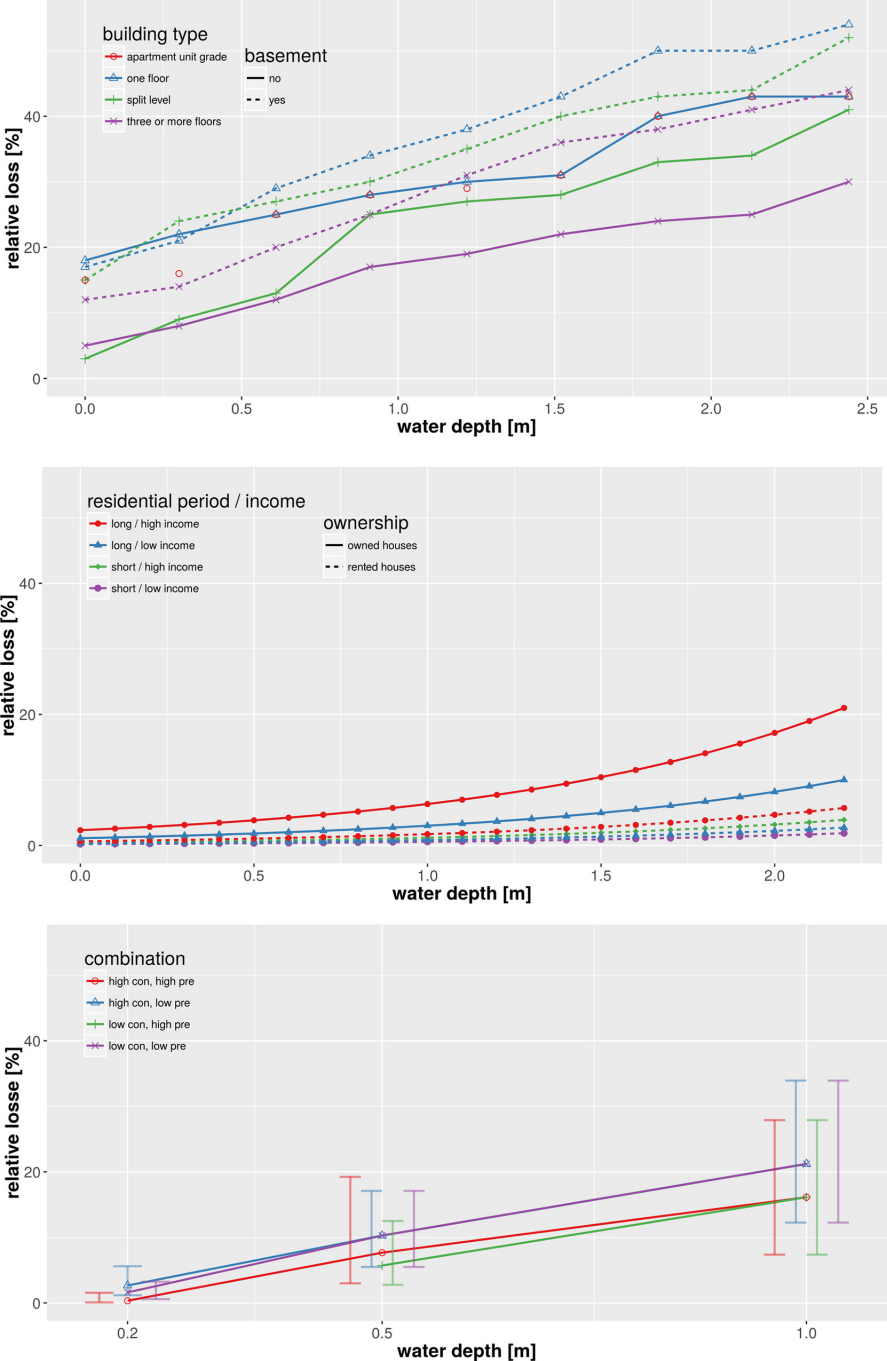
Harmonized flood loss models for residential buildings in dependence of water depth; top: HAZUS [43], middle: Zhai et al. [12], bottom: BN-FLEMOps [15].

To tackle an actual example, we illustrate the harmonization of flood loss models for the residential sector used in the previous section. Within this exercise we aim to compare these three models based on their common variables, following the second approach described above. The target space for harmonization is given by the relative building loss in percent in dependence on water depth in meters which is the only variable common to all three models. For the deterministic relative flood loss model HAZUS-MH [43] the harmonization is straightforward. As this model already provides relative building loss as a function of water depth, the only adjustment needed is to convert water depth from feet into meters. This model then provides a set of relative flood loss functions which differentiate residential buildings according to number of floors, presence of basement, and location within special flood hazard areas (Fig 8). The deterministic multi-variable damage model of Zhai et al. [12] provides absolute loss values as a function of water depth, ownership, residing period and income, and thus requires a two-tiered approach for harmonization. First, a set of uni-variable loss functions are obtained from the multi-variable model functions by determining the marginal functions which only depend on water depth. For this purpose, fixed values for house ownership (rental = 0 and owner = 1), residing period (short = 1 and long = 0) and income (low = 1 and high = 8) are used in combination with variable water depth values. The outcome is a set of damage functions depending on water depth which differentiate for house ownership, residing period and income level. Second, the absolute loss values are converted to relative values by dividing absolute values by the average single-family house price. Actually, different house prices or a price distribution should be taken into account e.g. for low and high income. For the sake of this exercise, however, we simply assume the average value of ¥72.11M suggested by Shimizu [62] for the year 2000 (Fig 8).

The harmonization of the multi-variable probabilistic model BN-FLEMOps [15] also requires two steps. First, a best estimate for relative loss needs to be inferred from the probability distribution. Second, the multi-variable joint probability distribution function has to be marginalized to obtain a uni-variable relation between relative damage and water depth. Within this illustrative example we use the median of the probability distribution as the best estimate for relative damage. The dimension of the multi-variable distribution is reduced by extracting separate functions for different combinations of values, the variables contamination ‘con‘ and precaution ‘pre‘ might take. This results in a set of damage functions each of them representing the expectation of relative flood damage for varying water levels given the specific combination of observations for the variables ‘con’ and ‘pre’. Fig 8 exemplifies the outcome of this procedure for the combination of ‘high contamination and high precaution’, ‘high contamination and low precaution’, ‘low contamination and high precaution’, and ‘low contamination and low precaution’. Each case is represented by a curve which depicts the expected relative damage, i.e. the median of the probability distribution, for three discrete water depth classes. The error bars show the inter-quartile range (IQR) of the probability distribution for the different combinations and water depth values and provide insight into uncertainty associated with the loss estimate.

**Fig 9:**
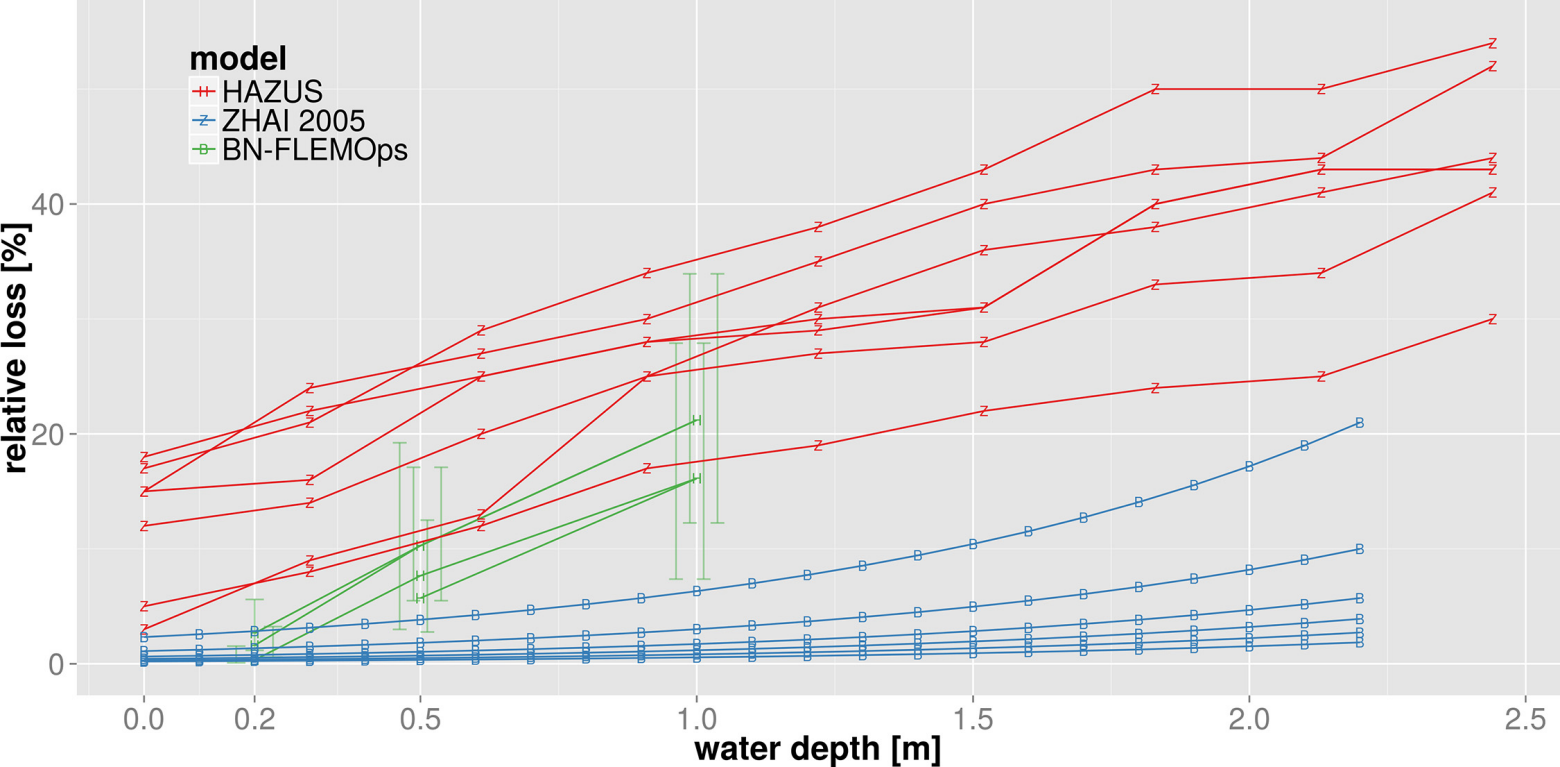
Compilation of harmonized flood loss models for residential buildings based on common variables.

Fig 9 shows all three models in the joint target space defined by relative building loss in percent and water depth in meters. The variability within HAZUS loss curves is due to building types and the presence of a basement, in the model of Zhai et al. [12] it is due to income class and residing period, and for BN-FLEMO [15] due to precaution and contamination. The information on different building types in different geographic regions is lost within the process of harmonization. Most striking is the large variability of relative loss estimates provided by the different models. This is consistent with previous findings [15, 32, 63] who showed the high sensitivity of model structure and loss function shape with regard to loss estimation. Further, our results show that model structural differences persist in spite of the adjustments made within harmonization. For example, the HAZUS functions show almost uniform loss gradients across the whole scale of water depths, whereas the functions of Zhai et al. [12] are based on a loss gradient which increases non-linearly with water depth. Note that the transformation of absolute loss estimates of Zhai et al. [12] to the harmonized relative loss estimates is clearly sensitive to the building value applied. For example, the variation of the average building value by ±50% gives a reduction of relative loss estimates by ⅔ and an increase by a factor of 2 respectively (not shown). Still, inter-model variability seems to be more important than intra-model variations due to differentiation of building type, number of floors, ownership structure, income and other characteristics. In this regard, the set of HAZUS loss functions shows the largest variability across the whole range of water depths. For the Zhai et al. [12] functions we observe that the spread of loss estimates increases with water depth. The BN-FLEMOps functions show the smallest amount of intra-model variability. The uncertainty range indicated by IQR is within the range of the alternative models HAZUS and Zhai et al. [12].

These findings emphasize the difficulties involved in assessing the suitability of a specific single model in comparison to other models within a harmonization framework. Our example illustrates the harmonization of only three alternative flood loss models for direct damage to residential buildings but the inventory contains various other models which could be included in a model suitability analysis framework with the aim of assessing the reasonability of model assumptions for application in risk assessments. The meta-data compiled in the inventory support the selection of suitable candidate models for benchmarking and comparison and thus may substantially reduce the effort required for model harmonization.

## Conclusions

The survey of 47 flood loss models, including nearly a thousand flood vulnerability relationships comprised in the inventory reveals model types of vastly heterogeneous characteristics. The large majority of models are based on a deterministic model concept. While they are still scarce, probabilistic models seem to be emerging in the literature. Most models are based, at least partly, on empirical data. Multi-variable models are widespread. They typically consist of a selection of uni-variable loss functions differentiated by building use, type, etc. From the inventory we see an almost equal share of relative and absolute loss estimation formats. Most models in the inventory refer to the residential sector; and clearly fewer models are available for other sectors like industry and infrastructure. Research efforts should be focused on these sectors if the objective were to perform risk assessments of public services. Validation seems not to be a standard step in model development, which is concerning. Quite a large share of papers that describe or propose flood loss models do not include any validation process or exhibits. Sometimes validation has been done later on in application studies or separately, in specific validation campaigns.

This flood loss model inventory constitutes a critical first step in the development of benchmarking and suitability analyses to support the evaluation and selection of models depending on the desired usage or regional focus in the insurance industry as well as in the flood risk community in general.

The harmonization of models for benchmarking and comparison is a tedious and difficult task that requires profound insight into the model structures, mechanisms and underlying assumptions. The most promising approach to achieve a quantitative comparison of disparate models probably relies on using variables that are common to the models. However, if the number of common variables is low the explanatory power of sophisticated multi-variable or probabilistic models is reduced, and thus the dispersion of loss estimates may become larger. This will make a relative quality assessment more challenging. This work has established the foundation to commence this harmonization exercise, critical for the validation of flood loss models in a common framework.

## Supporting Information

S1 Fig PRISMA 2009 Flow Diagram. (DOC)

S1 Table Loss model inventory. (PDF)

S2 Table PRISMA 2009 Checklist. (DOC)

S1 Text List of full text articles excluded. (DOCX)
