# Supplementary material for: A Review of Flood Loss Models as Basis for Harmonization and Benchmarking
Source: PLoS One. 2016 Jul 25;11(7):e0159791. doi: 10.1371/journal.pone.0159791 (PMC4959727; doi:10.1371/journal.pone.0159791)
Supplement: S1 Fig — (DOC) [file pone.0159791.s001.doc]

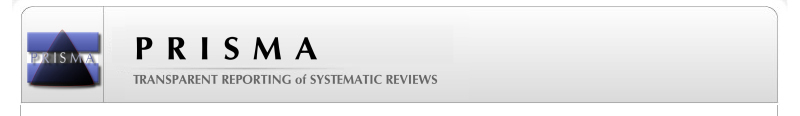
**PRISMA 2009 Flow Diagram**

**Screening**

**Included**

**Eligibility**

**Identification**

Records identified through database searching
(n =66)

Additional records identified through other sources
(n =25)

Records after duplicates removed
(n =89)

Records screened
(n =89)

Records excluded
(n =0)

Full-text articles assessed for eligibility
(n =89)

Full-text articles excluded, with reasons
(n =28)

Studies included in qualitative synthesis
(n =61)

Studies included in quantitative synthesis (meta-analysis)
(n =47)
