# Supplementary material for: A Review of Flood Loss Models as Basis for Harmonization and Benchmarking
Source: PLoS One. 2016 Jul 25;11(7):e0159791. doi: 10.1371/journal.pone.0159791 (PMC4959727; doi:10.1371/journal.pone.0159791)
Supplement: S1 Table — (PDF) [file pone.0159791.s002.pdf]

S1 Table: Loss model inventory

Supplement to Review of flood loss models as a basis for harmonization and benchmarking

geographical scope - continent	reference	model	domain	approach	model type	model concept	purpose of model	cost base	damage metric	geographical scope - country	geographical scope - region/ catchment	geographical scope - city	spatial resolution	unit of analysis	flood type	sector abbreviations see *	input variables abbreviations see **
Africa	de Villiers et al. (2007)	Standard residential flood damage functions for South African conditions (TEWA)	scientific paper	synthetic	multivariate	deterministic	total economic loss	depreciate costs	absolute	South Africa	Upington / Orange River (generalised version for other regions and catchment areas)	Upington	local	aggregated land use classes	fluvial	r_b, r_c	wd, bt, fsp, bv, bcont
Africa	Risi et al. (2013)	fragility curves	scientific paper	synthetic	multivariate	probabilistic	total economic loss	repair costs	absolute	Tanzania	Msimbazi River	Suna	local	individual objects	fluvial	r_b	wd, fv, rp, bfr
Asia	ADB (2010)	Asian Development Bank loss model (ADB)	report	synthetic	multivariate	deterministic	total economic loss	repair costs	relative	Bangladesh		Khulna	local	individual households	fluvial, water logging	r_b, c_b, i_b, a, i	wd, bt
Asia	Budiyono et al. (2015)	adapted version of the Damagescanner model (Aerts et al. 2008)	scientific paper	synthetic	multivariate	deterministic	economic loss		relative	Indonesia	Ciliwung River	Jakarta, Bangkok, Ho Chi Minh City, Manila	local	aggregated land use classes	fluvial	r_b, c_b, i_b	wd, fv, bt
Asia	Chang et al. (2008)	Geographically Weighted Regression Model (GWR)	scientific paper	combination	univariate	deterministic	total economic loss		absolute	Taiwan	Keelung River basin		regional	induvidual objects	fluvial	r_b	wd
Asia	Chau et al. (2015)	generalised simple damage function from Messner et al. (2007)	scientific paper	combination	multivariate	deterministic	total loss	replacement costs	relative	Vietnam	Quang Nam province	districts Dai Loc, Dien Ban, Duy Xuyen, Hoi An, Thang Binh, Phu Ninh, Que Son, Nui Thanh and Tam Ky	local	aggregated land use classes	fluvial	a	wd, rp, cropt
Asia	Dutta et al. (2003)		scientific paper	empirical	multivariate	deterministic	total economic loss	replacement costs	relative	Japan	Ichinomiya river basin, Chiba prefecture		regional	aggregated land use classes	fluvial	r_b, r_c, c_b, c_c, i_b, i_c, a, i	wd, id, tfl, bt, cmat, bcont, gps
Asia	Kim et al. (2012)	regional regression function	scientific paper	empirical	univariate	deterministic			absolute	South Korea		Seoul, Busan, Gwangju	local	aggregated land use classes	fluvial	r_b, c_b, i_b, pm_b, i	met, socec
Asia	Kobayashi et al. (2014)	Kyoto, Kobe University-SJNK economic loss estimation model (KKU-SJNK)	scientific paper	synthetic	univariate	deterministic	economic loss		relative	Japan	Osaka Prefecture for the Neyagawa River catchment	Neyagawa	local	individual objects	fluvial		wd
Asia	Su et al. (2009)		scientific paper	empirical	multivariate	deterministic	total economic loss		absolute	Taiwan		Taipei City	local	aggregated land use classes	fluvial	c_b, i_b	wd, bt, fsp
Asia	Yazdi&Neyshabouri (2012)	damage-elevation curves	scientific paper	empirical	multivariate	deterministic	total damage		relative	Iran	Kan basin		regional	aggregated land use classes	fluvial	r_b, r_c, c_b, a	wd, fv, bt, bcont, cropt
Asia	Zhai et al. (2005)		scientific paper	empirical	multivariate	deterministic			absolute, relative	Japan	Shin-Kawa River Basin	Nagoya city	regional	individual buildings	fluvial	r_b, r_c	wd, cmat, own, inco, res
Australia	Department of Natural Resources and Mines (NR&M) (2002)	ANUFlood	report	empirical	multivariate	deterministic			absolute	Australia			local	individual objects	fluvial	r_b, c_b	wd, fsp, bv
Australia	NRE (2000)	Rapid Appraisal Method (RAM)	report	combination	multivariate	deterministic	total economic loss		absolute	Australia	Victoria	Euroa, Traralgon, Wangaratta	local	individual objects	fluvial	a	id, tfl, cropt
Australia	WRM (2006)	WRM model	report	combination	multivariate	deterministic	total damages		absolute	Australia	Brisbane Valley	Brisbane City	local	individual buildings	fluvial	r_b, r_c, c_b, i_b, pm_b	wd, bt, nflo, fsp, bv
Central America	Vojinovic et al. (2008)		conference paper	combination	multivariate	deterministic	total economic loss		absolute	St Maarten			national	induvidual objects	fluvial	r_b, c_b, i_b	wd, bt, fsp
Europe	BMLFUW (2009)	Bundesministerium für Land- und Forstwirtschaft, Umwelt und Wasserwirtschaft flood loss model (BMLFUW)	report	empirical	univariate	deterministic	total economic loss		absolute	Austria			national	aggregated land use classes	fluvial	r_b	wd
Europe	Bremond (2011)	Évaluation de la Vulnérabilité Agricole (EVA)	scientific (PhD-Thesis)	empirical	multivariate	deterministic	total economic losses	depreciated/repair costs	absolute	France	Downstream Rhone		regional	individual objects	fluvial	a	wd, equ
Europe	Buck&Merkel (1999)	HOchWAsserSchäden (HOWAS)	report	empirical	multivariate	deterministic			absolute	Germany			regional	individual objects	fluvial	r_b, r_c	wd, bt, nflo, bcont
Europe	Förster et al. (2008); Thieken et al. (2008)	Methods for the evaluation of direct and indirect flood losses (MEDIS)	scientific paper	combination	multivariate	deterministic	total economic loss	replacement costs	relative	Germany	Elbe		local - regional	aggregated land use classes	fluvial, dam break	a, i	id, tfl, bv, cropt
Europe	Grahn&Nyberg (2014)	multiple linear regression model	scientific paper	empirical	multivariate	deterministic	insured damages		absolute	Sweden	lake Vänernand, lake Glafsfjorden		regional	individual buildings	lake floods	r_b	wd, id, dwf, int, nflo, age, prec, dam
Europe	Hallegatte et al. (2008)		report	empirical	multivariate	deterministic	insured assets		absolute	Denmark		Copenhagen	local	aggregated land use classes	coastal	r_b, r_c, c_b, c_c, i_b, i_c	wd, prec
Europe	Hess&Morris (1988)	annual flood loss	scientific paper	combination	multivariate	deterministic	total loss	replacement costs	absolute	England			regional	aggregated land use classes	fluvial	a	wd, tfl, cropt, repl
Europe	Huttenlau et al. (2010)	TYROL	scientific paper	empirical	multivariate	deterministic	total economic loss	replacement value, depreciated value	absolute	Austria	Tyrol		regional	individual objects	fluvial	r_b, c_b, i_b	wd, bt
Europe	Hydrotec (2002)	HWS-GIS	report	empirical	multivariate	deterministic	total economic losses		relative	Germany	Lippe	Lippstadt, Lippetal, Hamm, Werne, Bergkamen, Lünen, Haltern, Marl, Dorsten, Wesel	local	aggregated land use classes	fluvial	r_b, c_b, i_b, pm_b	wd, bt
Europe	IKSE (2003)	Internationale Kommission zum Schutz der Elbe (IKSE)	report	combination	multivariate	deterministic	total economic loss		relative	Germany	Elbe		regional	aggregated land use classes	fluvial	r_b, c_b, a, i	wd, bt, bcont
Europe	IKSR (2001)	International Commission for the Protection of the Rhine (ICPR)	report	combination	multivariate	deterministic	total economic losses	repair cost	relative	Switzerland, Germany, France, Netherlands	Rhine		regional	aggregated land use classes	fluvial	r_b, r_c, c_b, c_c, i_b, i_c, pm_b, a, i	wd, bt, equ
Europe	Klijn et al. (2007)	damage scanner model (DSM)	report	combination	multivariate	deterministic		replacement costs	relative	The Netherlands			regional	aggregated land use classes	fluvial, coastal	r_b, i_b, pm_b, a, i	wd, bt, cropt
Europe	Kreibich&Thieken (2008)	stage damage curves (linear, square-root, polynominal) (FLEMOps+ GHW)	scientific paper	empirical	multivariate	deterministic	total economic loss	replacement costs	relative	Germany	Elbe		regional	object-based	groundwater	r_b, r_c	wd, bt, bcont
Europe	Luino et al. (2009)	Boesio Stream model	scientific paper	empirical	univariate	deterministic	total economic loss	repair costs	relative	Italy	Boesio basin in the Lombardy Region		regional	aggregated land use classes	fluvial	r_b	wd
Europe	Maiwald&Schwarz (2010)	Earthquake Damage Analysis Center flood damage model (EDAC)	report	engineering	multivariate	deterministic			relative	Germany	Vereinigte Mulde, Zschopau, Freiberger Mulde		regional	individual objects, aggregated land use classes	fluvial	r_b	wd, nflo, cmat
Europe	Merz et al. (2013)	regression tree models	scientific paper	empirical	multivariate	deterministic	total economic loss		relative	Germany	Elbe, Danube		regional	object-based	fluvial	r_b	wd, id, fv, con, rp, bt, nflo, fsp, bq, bv, hs, inco, prec, exp, warn
Europe	MURL (2000)	Ministerium für Umwelt, Raumordnung und Landwirtschaft des Landes Nordrhein-Westfalen (MURL)	report	empirical	multivariate	deterministic	total economic loss	replacement costs	relative	Germany	Lower Rhine		regional	aggregated land use classes	fluvial	r_b, r_c, c_b, c_c, i_b, i_c, a, i, v	wd, bt, bcont, equ, gps
Europe	Notaro et al. (2014)	linear, polynomial 2-ord, exponential and power functions	conference paper	combination	multivariate	deterministic	total damage		absolute	Italy			regional	aggregated land use classes	fluvial	r_c, c_c, i_c, v	wd, gps
Europe	Penning-Rowsell et al. (2005)	Multi-coloured manual (MCM)	user manual	combination	multivariate	deterministic	total economic loss	depreciated value	absolute	England, Wales			national	aggregated land use classes	fluvial, coastal	r_b, r_c, c_b, c_c, i_b, i_c, pm_b	wd, id, bt, equ, gps
Europe	Prettenthaler et al. (2010)	absolute flood-damage function	scientific paper	empirical	multivariate	deterministic	expected damage	replacement costs	absolute	Austria	municipality of Dürnkrut		regional	individual buildings	dam breach	r_b	wd, con, nflo
Europe	Reese et al. (2003)	Mikroskalige Evaluation der Risiken in überflutungs-gefährdeten Küstenniederungen (MERK)	report	combination	multivariate	deterministic	total economic loss	replacement costs	relative	Germany	Coast of Schleswig-Holstein	St. Peter-Ording, Kaiser-Wilhelm Koog, Kiel, Fehmarn, Scharbeutz, Timmendorfer Strand	local	object-based	coastal	r_b, r_c, c_b, c_c, i_b, i_c, pm_b, i, v	wd, nflo, equ
Europe	Riha&Marcikova (2009)		conference paper	synthetic	multivariate	deterministic	total economic loss		relative	Czech Republic			regional	aggregated land use classes	fluvial	r_b	wd, id, cmat
Europe	Schröter et al. (2014)	Bayesian Network Flood Loss Model for the private sector (BN-FLEMOps)	scientific paper	empirical	multivariate	probabilistic			relative	Germany	Elbe, Danube		local	individual buildings	fluvial	r_b	wd, id, fv, con, rp, bt, nflo, fsp, bq, bv, bcont, hs, own, prec, exp, warn
Europe	Thieken et al. (2008), Kreibich et al. (2010), Elmer et al. (2010)	Flood Loss Estimation MOdel (FLEMO)	scientific paper	empirical	multivariate	deterministic	losses to different asset types		relative	Germany	Elbe, Danube	Döbeln, Eilenburg, Grimma	local	aggregated land use classes	fluvial	r_b, c_b	wd, con, ri, bt, bq, bcont, sicom, seccom, equ, gps, prec
Europe	Torterotot (1993)		scientific (PhD-Thesis)	empirical	bulk-model (roads), univariate (residential buildings)	deterministic	total economic losses	depreciated/repair costs	relative	France			national	aggregated land use classes	fluvial	r_b, r_c, i	wd, fv, nflo, bcont, prec, warn
Europe	Toth et al. (2008)		report	synthetic	multivariate	deterministic	total economic loss	depreciated costs	relative	Hungary	Körös corner flood area		regional	aggregated land use classes	fluvial	r_b, r_c, c_b, c_c, i_b, i_c, a	wd, bt, nflo, cmat, age, equ, gps, repl
Europe	Totschnig et al. (2011)	fluvial sediment transport model	scientific paper	empirical	univariate	deterministic	total damage	reconstruction value	relative	Austria	Tyrol, Carinthia		local	individual buildings	fluvial, sediment transport	r_b	wd
Europe	Vanneuville et al. (2006)	Flemish	report	synthetic	multivariate	deterministic			relative	Belgium			regional	aggregated land use classes	fluvial	r_b, i_b, pm_b, a, i	wd, bt, cropt
North America	FEMA (2009); Scawthorn et al. (2006)	HAZUS-MH	user manual	combination	multivariate	deterministic	total economic loss	replacement costs	relative	USA			national, regional	individual buildings, aggregated land use classes	riverine, coastal	r_b, r_c, c_b, c_c, i_b, i_c, pm_b, a, (i), v	wd, tfl, bt, nflo, bcont, equ, gps, cropt
North America	Grigg and Helweg (1975)		scientific paper	combination	bulk-model	deterministic	total economic losses	depreciated/repair costs	relative	USA			national	aggregated land use classes	fluvial	r_b, c_b, i_b, pm_b, i	wd, bt
North America	Pistrika&Jonkman (2010)		scientific paper	empirical	multivariate	deterministic	total economic loss	repair costs	relative	USA	Mississippi River	New Orleans	local	aggregated land use classes	fluvial, levee breach	r_b	wd, fv

* r_b=residential building, r_c=residential content, c_b=commercial building, c_c=commercial content, i_b=industrial building, i_c=industrial content, pm_b=public and municipal building, a=agriculture, i=infrastructure, v=vehicles

** wd=water depth, id=inundation duration, fv=flow velocity, con=contamination, rp=return period, tfl=time of flood event, ri=recurrence interval, dwf=distance between damaged object and water front, met= meteorological data, int=intercept, bt=building type, nflo=number of floors/basement/cellar, fsp=floor space, cmat=construction material, age=age of building, bq=building quality, bv=building value, bfr=building fragility, bcont=building content/inventory, socec=socio-economic factors, hs=household size, own=ownership (e.g. rental, private), inco=monthly net income, res=residing period, sicom=size of company (nr. employees), seccom=sector of company, equ=equipment, gps=goods/products/stock/vehicles, cropt=crop type, prec=precaution, exp=flood experience, warn=early warning, repl=cost of replacement feed, additional costs incurred (+) or saved (-), dam=damage to building (no building damage or building damage)
