# Supplementary material for: A Review of Flood Loss Models as Basis for Harmonization and Benchmarking
Source: PLoS One. 2016 Jul 25;11(7):e0159791. doi: 10.1371/journal.pone.0159791 (PMC4959727; doi:10.1371/journal.pone.0159791)
Supplement: S2 Table — (DOC) [file pone.0159791.s003.doc]

| **Section/topic** | **#** | **Checklist item** | **Reported on page #** |
| --- | --- | --- | --- |
| **TITLE** | | |  |
| Title | 1 | Identify the report as a systematic review, meta-analysis, or both. | Title includes explicitly that it is a “review” |
| **ABSTRACT** | | |  |
| Structured summary | 2 | Provide a structured summary including, as applicable: background; objectives; data sources; study eligibility criteria, participants, and interventions; study appraisal and synthesis methods; results; limitations; conclusions and implications of key findings; systematic review registration number. | Abstract briefly describes elements of the PRISMA flow diagram. No participants and interventions are discussed since this is not applicable to the exercise.GF |
| **INTRODUCTION** | | |  |
| Rationale | 3 | Describe the rationale for the review in the context of what is already known. | In the last section of the introduction, we describe that the rationale is to aggregate the information to give a broad perspective of the current scope of knowledge, see p 5, ll 99-105. |
| Objectives | 4 | Provide an explicit statement of questions being addressed with reference to participants, interventions, comparisons, outcomes, and study design (PICOS). | It is not really applicable to our study but we do specifically mention that the main question we query from the studies included in the review is that they include the mathematical formulations of the damageability relationships which we set out to catalog, see p. 5, l. 103. |
| **METHODS** | | |  |
| Protocol and registration | 5 | Indicate if a review protocol exists, if and where it can be accessed (e.g., Web address), and, if available, provide registration information including registration number. | N/A: There is no protocol on-line but the Methods section explicitly describes the identification process followed by authors, see pp 6-7. |
| Eligibility criteria | 6 | Specify study characteristics (e.g., PICOS, length of follow-up) and report characteristics (e.g., years considered, language, publication status) used as criteria for eligibility, giving rationale. | This is also specified in the Methods section, see p. 8 ll. 157 – 158. |
| Information sources | 7 | Describe all information sources (e.g., databases with dates of coverage, contact with study authors to identify additional studies) in the search and date last searched. | All the sources used are specified in the Methods sections, see p.7, ll. 143-1144. References are collected in the Reference section along with dates and addresses for electronic sources, see pp. 30-34. |
| Search | 8 | Present full electronic search strategy for at least one database, including any limits used, such that it could be repeated. | The Methods section describes the protocol used for the search in clearly identifiable steps, see pp. 7-8, ll. 143-158. |
| Study selection | 9 | State the process for selecting studies (i.e., screening, eligibility, included in systematic review, and, if applicable, included in the meta-analysis). | The Methods section specifies the process followed for selecting studies, see p 13, ll. 185 – 195. |
| Data collection process | 10 | Describe method of data extraction from reports (e.g., piloted forms, independently, in duplicate) and any processes for obtaining and confirming data from investigators. | The extraction process in our case is relatively simple since we are interested only in the mathematical formulations, which we have collected from the sources and catalogued according to the metrics described in the paper. No confirmations were pursued with the original authors of the sources; see p. 13, ll. 185 – 195. |
| Data items | 11 | List and define all variables for which data were sought (e.g., PICOS, funding sources) and any assumptions and simplifications made. | Categories and attributes are listed in Table 1, see pp. 9-13. |
| Risk of bias in individual studies | 12 | Describe methods used for assessing risk of bias of individual studies (including specification of whether this was done at the study or outcome level), and how this information is to be used in any data synthesis. | N/A: The Paper does not review outcomes but functional assumptions therefore bias is not present. We simply collect the mathematical descriptions presented in the sources |
| Summary measures | 13 | State the principal summary measures (e.g., risk ratio, difference in means). | The main metric used is simply the count of models grouped into the categories listed in Table 1 (Methods section) and illustrated in Fig 1 in Observations. |
| Synthesis of results | 14 | Describe the methods of handling data and combining results of studies, if done, including measures of consistency (e.g., I^2^) for each meta-analysis. | N/A: Combination of results (models) is beyond the scope of this review. |

Page 1 of 2

| **Section/topic** | **#** | **Checklist item** | **Reported on page #** |
| --- | --- | --- | --- |
| Risk of bias across studies | 15 | Specify any assessment of risk of bias that may affect the cumulative evidence (e.g., publication bias, selective reporting within studies). | N/A: Please, see checklist item 12 |
| Additional analyses | 16 | Describe methods of additional analyses (e.g., sensitivity or subgroup analyses, meta-regression), if done, indicating which were pre-specified. | N/A: Please, see checklist item 14 |
| **RESULTS** | | |  |
| Study selection | 17 | Give numbers of studies screened, assessed for eligibility, and included in the review, with reasons for exclusions at each stage, ideally with a flow diagram. | Please, see attached PRISMA Flow Diagram. A paragraph has also been added to the Observations section with the flow diagram description, see p 13, ll. 185 – 195. |
| Study characteristics | 18 | For each study, present characteristics for which data were extracted (e.g., study size, PICOS, follow-up period) and provide the citations. | Please, see Table 1 for characteristics collected in the review exercise as well as the Reference section for citations and explicit source information. |
| Risk of bias within studies | 19 | Present data on risk of bias of each study and, if available, any outcome level assessment (see item 12). | N/A: Please, see checklist item 12 |
| Results of individual studies | 20 | For all outcomes considered (benefits or harms), present, for each study: (a) simple summary data for each intervention group (b) effect estimates and confidence intervals, ideally with a forest plot. | N/A: Please, see checklist item 12 |
| Synthesis of results | 21 | Present results of each meta-analysis done, including confidence intervals and measures of consistency. | N/A: Please, see checklist items 13 and 14 |
| Risk of bias across studies | 22 | Present results of any assessment of risk of bias across studies (see Item 15). | N/A: Please, see checklist item 12 |
| Additional analysis | 23 | Give results of additional analyses, if done (e.g., sensitivity or subgroup analyses, meta-regression [see Item 16]). | N/A: Please, see checklist items 12 - 14 |
| **DISCUSSION** | | |  |
| Summary of evidence | 24 | Summarize the main findings including the strength of evidence for each main outcome; consider their relevance to key groups (e.g., healthcare providers, users, and policy makers). | N/A: Since our exercise concentrates on the collection of functional forms, no outcomes are given more or less emphasis. Our findings regarding the qualitative distribution of characteristics are discussed in sections Observations and Conclusions, see pp.13 - 19 and pp-29-30 |
| Limitations | 25 | Discuss limitations at study and outcome level (e.g., risk of bias), and at review-level (e.g., incomplete retrieval of identified research, reporting bias). | See p.7, ll. 140 – 143 and p. 8, ll. 159 – 164. |
| Conclusions | 26 | Provide a general interpretation of the results in the context of other evidence, and implications for future research. | Authors provide general interpretation of results and observations both in sections Observations and Conclusions. |
| **FUNDING** | | |  |
| Funding | 27 | Describe sources of funding for the systematic review and other support (e.g., supply of data); role of funders for the systematic review. | Guy Carpenter and Company Ltd supported parts of this research. However the funders had no role in the review design, data collection and decision to publish. |

*From:*  Moher D, Liberati A, Tetzlaff J, Altman DG, The PRISMA Group (2009). Preferred Reporting Items for Systematic Reviews and Meta-Analyses: The PRISMA Statement. PLoS Med 6(6): e1000097. doi:10.1371/journal.pmed1000097

For more information, visit: **www.prisma-statement.org**.

Page 2 of 2
