# Supplementary material for: A Review of Flood Loss Models as Basis for Harmonization and Benchmarking
Source: PLoS One. 2016 Jul 25;11(7):e0159791. doi: 10.1371/journal.pone.0159791 (PMC4959727; doi:10.1371/journal.pone.0159791)
Supplement: S1 Text — (DOCX) [file pone.0159791.s004.docx]

**S1 Text: Excluded references**

Australia and Bureau of Transport Economics: Economic costs of natural disasters in Australia, Bureau of Transport Economics, Canberra., 2001.

Ballesteros-Cánovas, J. A., Sanchez-Silva, M., Bodoque, J. M. and Díez-Herrero, A.: An Integrated Approach to Flood Risk Management: A Case Study of Navaluenga (Central Spain), Water Resources Management, 27(8), 3051–3069, doi:10.1007/s11269-013-0332-1, 2013.

Blong, R.: A new damage index, Natural hazards, 30(1), 1–23, 2003.

Boettle, M., Kropp, J. P., Reiber, L., Roithmeier, O., Rybski, D. and Walther, C.: About the influence of elevation model quality and small-scale damage functions on flood damage estimation, Natural Hazards and Earth System Science, 11(12), 3327–3334, doi:10.5194/nhess-11-3327-2011, 2011.

Booij, M. J.: Flood damage assessment and modelling in the Red River basin in Vietnam, in International Workshop on Flood Controls Decision Support Systems (Flocods), Vietnam., 2004.

Brémond, P. and Grelot, F.: Comparison of a systemic modelling of farm vulnerability and classical methods to appraise flood damage on agricultural activities, in 11th Biennal Conference of the International Society for Ecological Economics (ISEE) Advancing Sustainability in a Time of Crisis, p. 20 p., 2010.

del Carmen Silva-Aguila, N., López-Caloca, A. and Silván-Cárdenas, J. L.: Damage estimation on agricultural crops by a flood, in SPIE Remote Sensing, p. 81740D–81740D–15, International Society for Optics and Photonics., 2011.

De Jonge, T., Kok, M. and Hogeweg, M.: Modelling floods and damage assessment using GIS, IAHS PUBLICATION, 299–306, 1996.

Department of Environment and Climate Change (DECCW), Floodplain Risk Management Guideline, New South Wales, Australia, p. 10, 2007

Hall, J. W., Sayers, P. B. and Dawson, R. J.: National-scale assessment of current and future flood risk in England and Wales, Natural Hazards, 36(1-2), 147–164, 2005.

Handmer, J. W.: ANUFLOOD in New Zealand: Part 2, Background to flood loss measurement, Centre for Resource and Environmental Studies, Australian National University Canberra, 1986.

Lekuthai, A. and Vongvisessomjai, S.: Intangible flood damage quantification, Water Resources Management, 15(5), 343–362, 2001.

Maiwald, H. and Schwarz, J.: Schadensmodelle für extreme Hochwasser - Teil 1: Modellbildung und Validierung am Hochwasser 2002, Bautechnik, 91(3), 200–210, doi:10.1002/bate.201300101, 2014.

Markau, H.-J. and Reese, S.: Naturgefahren und Risikobetrachtung im schleswig-holsteinischen Küstenraum, DIE KÜSTE, Kuratorium für Forschung im Küsteningenieurwesen (Hrsg.)/Heft, 67, 3–22, 2003.

Meyer, V. and Messner, F.: National flood damage evaluation methods–A Review of Applied Methods in England, The Netherlands, The Czech Republic and Germany, FLOODsite consortium, GOCE-CT-2004-505420., 2005.

Meyer, V., Scheuer, S. and Haase, D.: A multicriteria approach for flood risk mapping exemplified at the Mulde river, Germany, Natural Hazards, 48(1), 17–39, doi:10.1007/s11069-008-9244-4, 2009.

de Moel, H. and Aerts, J. C. J. H.: Effect of uncertainty in land use, damage models and inundation depth on flood damage estimates, Natural Hazards, 58(1), 407–425, doi:10.1007/s11069-010-9675-6, 2011.

Muir Wood, R., Drayton, M., Berger, A., Burgess, P. and Wright, T.: Catastrophe loss modelling of storm-surge flood risk in eastern England, Philosophical Transactions of the Royal Society A: Mathematical, Physical and Engineering Sciences, 363(1831), 1407–1422, doi:10.1098/rsta.2005.1575, 2005.

Pingel, N. and Watkins Jr, D.: Multiple flood source expected annual damage computations, Journal of Water Resources Planning and Management, 136(3), 319–326, 2009.

Riddell, K. and Green, C.: Flood and coastal defence project appraisal guidance: economic appraisal, Ministry of Agriculture, Food and Fisheries (MAFF), 1999.

Rivers Agency, Preliminary Flood Risk Assessment and Methodology for the Identification of Significant Flood risk Areas, Belfast, p. 126, 2011

Sayers, P., Hall, J., Dawson, R. and Rosu, C.: Risk assessment of flood and coastal defences for strategic planning (RASP)–a High Level Methodology, 2002.

Ten Veldhuis, J. A. E. and Clemens, F.: Flood risk modelling based on tangible and intangible urban flood damage quantification, Water Science and Technology, 62(1), 189, 2010.

Vogel, K., Riggelsen, C., Merz, B., Kreibich, H. and Scherbaum, F.: Flood damage and influencing factors: a Bayesian network perspective, in 6th European Workshop on Probabilistic Graphical Models (PGM 2012), University of Granada, Granada, Spain., 2012.

Wagemaker, J., Leenders, J. and Huizinga, J.: Economic valuation of flood damage for decision makers in the Netherlands and the Lower Mekong River Basin, in 6th Annual Mekong Flood Forum, pp. 27–28., 2008.

White, G. F.: Human adjustment to floods: a geographical approach to the flood problem in the United States, University of Chicago Chicago., 1945.

Worley & Parsons, Floodplain Risk Management, Newcastle, p 175, 2009

Zhang, B., Di, L., Yu, G., Shao, Y., Shrestha, R. and Kang, L.: A Web service based application serving vegetation condition indices for Flood Crop Loss Assessment, in Agro-Geoinformatics (Agro-Geoinformatics), 2013 Second International Conference on, pp. 215–220, IEEE., 2013.
